# Notes on the Dentition of the Mexican Hairless Dogs

**Published:** 1890-04

**Authors:** James A. Waugh

**Affiliations:** U. S. Army


### NOTES ON THE DENTITION OF MEXICAN HAIRLESS DOGS.

By James A. Waugh, V.S.,
U. S. Army.

A careful inspection of a number of Mexican hairless dogs, observed during my travels in Arizona, California, New Mexico, Texas and Mexico, convinces me that the dentition of these dogs varies considerably, and in many respects differs from that of ordinary domestic dogs. The teeth are generally irregular in number and relative position in the jaws. They have no tubercles or trefoils on the table surfaces of the incisor teeth. There are no canine teeth, or tushes, in either jaw. However, I have met with some half-breeds that had canine teeth in the upper or the lower jaw, but found no cases with those teeth in both jaws. The molars vary in number and relative position in different animals. The development of the adult dentition is much slower and later than in other classes of dogs. These facts may prove of some interest to naturalists and comparative anatomists. The following brief outline of the dental formulas of two small Mexican hairless dogs owned by the writer serve to illustrate the dentition of these curious little animals.

*a*. Mexican hairless dog, male ; aged 2 years ; weight, twelve pounds. Superior incisors, five ; no canine teeth ; superior molars, two on each side, one large molar and one very small molar behind the large one. Inferior incisors two ; no canine teeth ; inferior molars, two on each side, one large molar and one very small molar behind the large one. Total number of teeth, 15.

*b.* Mexican hairless dog, female; aged 2 years; weight, eleven pounds. Superior incisors, four in front and one on right side midway between front teeth and the molars; no canine teeth; superior molars, two on each side, one small molar and one large molar behind the smaller one. Inferior incisors, seven in front and two on each side midway between the front teeth and the molars; no canine teeth; inferior molars, two on each side, one large molar and one small molar behind the large one. Total number of teeth, 24.

